# Mechanical Properties and Thermal Conductivity of Thermal Insulation Board Containing Recycled Thermosetting Polyurethane and Thermoplastic

**DOI:** 10.3390/polym13244411

**Published:** 2021-12-16

**Authors:** Ping He, Haoda Ruan, Congyang Wang, Hao Lu

**Affiliations:** College of Mechanical and Electrical Engineering, Anhui Jianzhu University, Hefei 230601, China; rhd@stu.ahjzu.edu.cn (H.R.); wangcongyang@stu.ahjzu.edu.cn (C.W.); luhao6083@sina.cn (H.L.)

**Keywords:** mechanochemical method, recycled polyurethane foam, orthogonal test, tensile strength, thermal conductivity

## Abstract

This study used a mechanochemical method to analyze the recycling mechanism of polyurethane foam and optimize the recycling process. The use of mechanochemical methods to regenerate the polyurethane foam powder breaks the C–O bond of the polyurethane foam and greatly enhances the activity of the powder. Based on orthogonal test design, the mesh, proportion, temperature, and time were selected to produce nine recycled boards by heat pressing. Then, the influence of four factors on the thermal conductivity and tensile strength of the recycled board was analyzed. The results show that 120 mesh polyurethane foam powder has strong activity, and the tensile strength can reach 9.913 Mpa when it is formed at 205 °C and 40 min with 50% PP powder. With the help of the low thermal conductivity of the polyurethane foam, the thermal conductivity of the recycled board can reach 0.037 W/m·K at the parameter of 40 mesh, 80%, 185 °C, 30 min. This research provides an effective method for the recycling of polyurethane foam.

## 1. Introduction

Polyurethane is widely used in the construction industry, automobile industry, coatings, and clothing applications, because of its good stability, corrosion resistance, low density, and thermal conductivity [1]. Therefore, the production of polyurethane is also increasing. At present, the annual output of polyurethane is close to 30 million tons, accounting for 7.9% of the total output of plastics. It is the fifth most used polymer in the world [2]. Polyurethanes are generally divided into the following categories: flexible foams, rigid foams, and shells (coatings, adhesives, sealants, elastomers), which are used for the different applications shown in Table 1 [3,4,5,6].

In the process of production and consumption, a large number of polyurethane foam wastes have appeared. Due to the small pile-up density (about 30 kg/m^3^) and difficulty in natural degradation, polyurethane foam has caused serious environmental problems [7]. Many countries are researching biodegradable polyurethane foam, but the high price makes the traditional polyurethane foams cannot be replaced in a short time [8,9] Therefore, how to properly handle polyurethane foam waste is worth studying.

The treatment methods of polyurethane foam waste are landfill, incineration, and recovery [10,11]. The proportion of landfill waste can be as high as 50%. Because of the damage to the ecology and the environment, and the continuous depletion of oil reserves, many countries restrict or even prohibit the landfill of polymer waste [2]. Incineration, as another treatment method of polyurethane foam, occupies an important position. Incineration uses polyurethane waste as fuel to recover energy. In fact, polyurethane combustion can provide the same amount of heat as coal by weight [7]. However, flame retardants are added to many polyurethane foams, which greatly hinders the combustion of polyurethane. The incomplete combustion of polyurethane will produce toxic gases (such as CO, NOx) and pollute the atmosphere. Therefore, recycling will become the best way to deal with polyurethane foam.

After the polyurethane foam is cured, it cannot be reshaped by heating it again. The good performance of polyurethane foam makes recycling more difficult. At present, there are two methods to recycle waste polyurethane foam: physical recycling and chemical recycling [12,13].

The physical recycling method does not change the chemical structure. The polyurethane foam is broken into particles or powders, which can be directly used as filler or reshaped with adhesives [14]. Nowadays, the physical recycling method of polyurethane foam has been widely used. Yang et al. [15] crushed rigid polyurethane foam into particles to enhance the mechanical properties of rigid polyurethane foam (PUF) and phenolic foam (PF). The results show that when the particle polyurethane foam (PPU) content is 5 wt%, the compressive strength of PUF and PF has an increase of nearly 20%. Gama et al. [16] reported that PUF waste particles can be mixed with MDI and then molded at 100–200 °C and 30–200 bar pressure. The product of this method has been useful as insulation panels, carpets, and furniture. Moon et al. [17] use low-temperature pulverization to pulverize flexible polyurethane foam into powder. The polyurethane foam powder is treated by ultrasonic, and the original polyurethane foam is added to prepare mixed PUF. The results show that the car seat cushion made of mixed PUF has higher comfort than pure PUF and reduces the hardness and hysteresis loss. The physical recycling method is simple in operation and low cost, but its application range is limited, and its potential has not been extensively developed.

Chemical recycling methods, also known as raw material recovery, include alcoholysis, hydrolysis, glycolysis, acidolysis, etc. [18,19], which degrade polyurethane foam into oligomers and smaller molecules. The raw materials recovered by the chemical method can be used in new polyurethane foam or other products. Valle et al. [20] used castor oil to successfully decompose flexible polyurethane foam waste. The results show that increasing the concentration of Decomposed polyurethane (DP) will increase the elongation at break, reduce the tensile strength and the cell size. Heiran et al. [21] used different glycols and catalysts for the glycolysis of waste polyurethane foam. Parameters such as temperature and material ratio are determined. The recovered raw materials can be used to prepare new polyurethanes and be used in boiler insulation and protective coatings. Gama et al. [22] depolymerized flexible polyurethane foam with succinic acid to obtain recycled polyol. The recycled polyol will replace part of the original polyol to produce polyurethane foam. The results show that 30% recycled polyol has no obvious effect on the morphology and density of the polyurethane foam. The chemical recycling method follows the principle of degradation and is the best method for recycling polyurethane foam in theory. However, the process is complicated, and the separation and purification process are very expensive, which is difficult for industrial application.

Mechanochemistry is based on the physical method and accumulates mechanical energy and thermal energy, through long-term mechanical force action to make solid reactants react chemically without solvent and change the chemical structure of substances [23,24]. Although the thermosetting plastics cannot be reduced to raw materials by using the mechanochemical method, such as the chemical recovery method, it can interrupt the network crosslinking structure of thermosetting plastics, reducing the crosslinking degree and improving the activity of recycled powder. Hu et al. [25] used the mechanochemical method to recover thermosetting phenolic resin, and the tensile strength of the recycled material could reach 8.13 Mpa.

**Figure 1:**
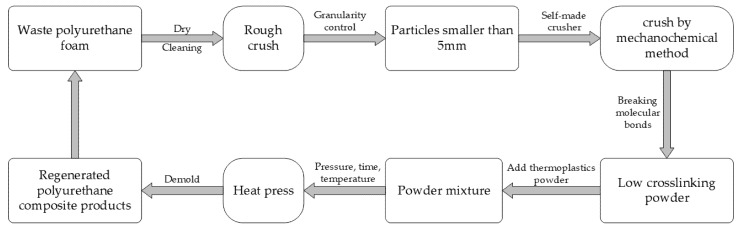
Recovery process of polyurethane foam by mechanochemical method.

In summary, the mechanical method is feasible for recycling thermosetting plastics, but it is mainly focused on the mechanical properties of recycled materials, which is undoubtedly a waste for polyurethane foam with high thermal insulation capacity. This research is an attempt to recycle polyurethane foam and make it into an insulation material that can be used in buildings. Mechanochemical method was used to recover polyurethane foam as filler, recycled polypropylene as the matrix, without adding any other adhesive, only change the polyurethane particle size, proportion, and heat pressing parameters, and the thermal conductivity and tensile strength of the product were evaluated. The recovery process of polyurethane foam by the mechanochemical method is shown in Figure 1.

## 2. Materials and Methods

### 2.1. Materials

**Figure 2:**
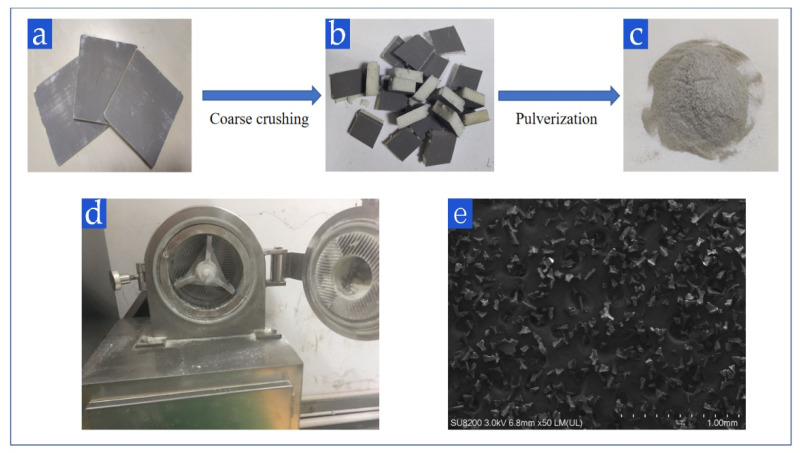
Appearance of (**a**) Waste PUF board, (**b**) PUF pieces, (**c**) PUF powder, (**d**) self-made crusher, and (**e**) microscopic morphology of PUF powder.

The waste polyurethane foam used in this study is rigid polyurethane insulation board (Aoyang Insulation Material Corp., Langfang, China). As shown in Figure 2, the outer side of this board is a fireproof layer composed of non-woven fabric and inorganic paste, and the middle is rigid polyurethane foam. This board is the most commonly used type of building insulation material in China. The matrix material is recycled polypropylene (ZhongLian Plastic Corp., Dongguan, China).

### 2.2. Experiment Process

#### 2.2.1. PUF Crushing Process

The crushing of the waste polyurethane foam is carried out in a self-made crusher specially designed for long-term crushing in the laboratory. To fit the actual recovery conditions, the fireproof layer was retained. It can enhance the strength of the recycled broad and improve the economic benefit. First, the polyurethane foam was manually cut into small pieces of 2 square centimeters, and then rough crushed into particles smaller than 5 mm, last, crushed into low crosslinking powder with the self-made crusher. As shown in Figure 2d, the self-made crusher is equipped with three sets of cutter teeth and two grinding discs. There are shear force, grinding force, extrusion force, and other mechanical forces in the grinding process. As the material is pulverized and heat energy accumulates, the network structure of polyurethane foam is broken and active groups are formed. The speed of the crusher is set to 1500 r/min, and the crushing time is 40 min. This crushing condition was obtained by previous studies in the laboratory [26]. Faster speed and longer time will enhance the crushing effect, but the mechanical energy consumption is greatly increased, and the efficiency is lower. At 1500 r/min and 40 min, polyurethane foam can be effectively degraded and has the highest cost performance. Polyurethane foam powder is shown in Figure 2c.

#### 2.2.2. Heat Press Process

**Figure 3:**
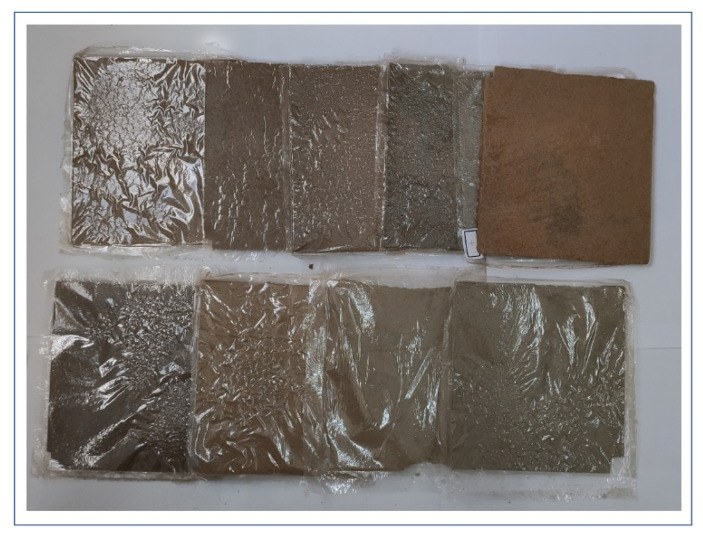
Recycled boards formed by heat press.

The polyurethane foam powder was molded with the flat vulcanizing machine XLB350X (Qicai Hydraulic Machinery Corp., Shanghai, China). To facilitate demolding, a layer of PET film is laid on the bottom of the mold. The melting point of PET is above 250 °C, which can prevent the melt from bonding with the mold. Mix the waste polyurethane foam powder and recycled PP material evenly, lay the mixture in the mold, put another layer of PET film on the powder, cover the press mold. Preheat mold at 175 °C for 10 min before each experiment. Then the heat pressing is carried out at the temperature and time in the table. After the heat pressing, the exhaust is released for 10 min, and then kept warm for 10 min. Finally, remove the mold and cool it to room temperature before demolding. The size of the board is 150 × 150 × 5 mm^3^, as shown in Figure 3.

### 2.3. Performance Testing

#### 2.3.1. PUF Powder Testing

The distribution of polyurethane foam powder was determined by laser particle size analyzer BT-9300ST (Bettersize Instruments Crop., Dandong, China). Distilled water and sodium pyrophosphate were added to the powder to make a suspension, and the powder was dispersed by ultrasonic for 3 min. The cycle speed during the test was 1600 rpm, and the average value was taken for 6 tests.

The Fourier transform infrared spectrometer FTIR-850 (GangDong Sci&Tech Ltd., Tianjin, China) was used to study the molecular structure changes of polyurethane foam powders. Three meshes of powders (40, 80, 120) were added into potassium bromide and made into press sheets, which were determined by 32 scanning times.

#### 2.3.2. Scanning Electron Microscope (SEM)

Scanning electron microscope EVO-18 (Carl Zeiss AG, Oberkochen, Baden-Württemberg, Germany) was used to observe the microstructure of polyurethane powder and recycled board. Considering the low electrical conductivity of polyurethane, the recycled boards were cut into 5 × 5 × 5 mm^3^ samples and coated with gold under vacuum. The acceleration voltage is selected as 20 kV, which can satisfy the analysis of most elements. Compared with the lower acceleration voltage, 20 kV can obtain a higher resolution and help us observe the composition information inside the sample.

#### 2.3.3. Thermal Conductivity Testing

Thermal conductivity has always been considered as the main parameter related to the practical application of polyurethane foam. Heat flow meter apparatus DRPL-III (XiangYi, Instrument Co., Ltd., Xiangtan, China) was used to detect the thermal conductivity of nine boards. Based on the ISO 8301 standard, select Two heat flow meter configurations, set the cold surface to 25 °C, the hot surface to 40 °C, and the pressure to 80 N. Thermal conductivity is calculated according to Formula (1). The experiments were repeated three times for each sample to obtain an average value. The samples measured for thermal conductivity were polished and refined to reduce thermal contact resistance.

(1) $$\lambda =0.5\left(f_{1}e_{1}+f_{2}e_{2}\right)\frac{d}{\Delta T}$$

where:

*λ* = thermal conductivity (W·m^−1^·K^−1^)
*f* = calibration factor (W·m^−2^·V^−1^)
*e* = heat flow meter output (V)
*d* = average specimen thickness (m)

#### 2.3.4. Tensile Strength Testing

Materials testing System AGS-X (Shimadzu Corp., Kyoto, Japan) was used to test the tensile strength of recycled boards, based on the International Organization for Standardization (ISO) 527 and ASTM D638. the recycled boards were made into standard-size samples (width 10 mm, gage length 50 mm), the test speed was set to 1 mm/min, and the maximum load that the sample could bear was measured. The experiment was repeated three times to determine the tensile strength.

## 3. Results and Discussion

The main purpose of this work is to develop a kind of recycled sheet with better mechanical properties and lower thermal conductivity. Thus, this can be viewed as an optimization problem with two objectives. The optimization process is mainly divided into the following parts:

(1) Analyze the crushing effect of polyurethane foam powder;
(2) Design and complete the orthogonal test;
(3) Take the thermal conductivity and tensile strength as the response values, analyze the influence of factors on them;
(4) Multi-objective optimization selection of recycled board.

### 3.1. Analysis of Crushing Effect

#### 3.1.1. Particle Size Distribution of PUF Powder

**Figure 4:**
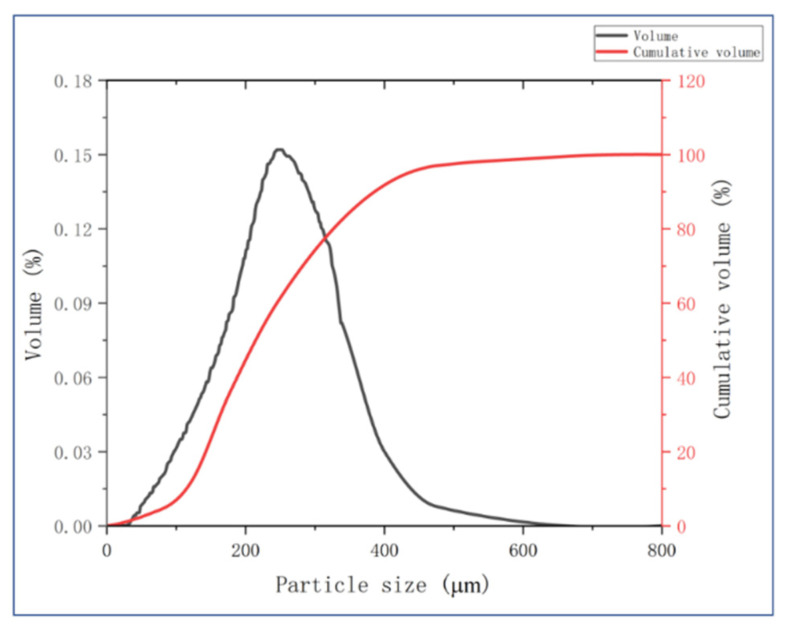
The particle size distribution of PUF powder.

Figure 4 showed the size distribution of the pulverized PUF powder. The average size of PUF powder is 245 µm, which will not greatly affect the board forming and can retain certain thermal insulation performance. The particle size distribution is: 52.89% = 177–420 µm, 22.83% = 180–125 µm; 8.74% = 75–125 µm; 4.21% < 75 µm.

#### 3.1.2. FTIR (Fourier Transform Infrared Spectroscopy) Analysis

**Figure 5:**
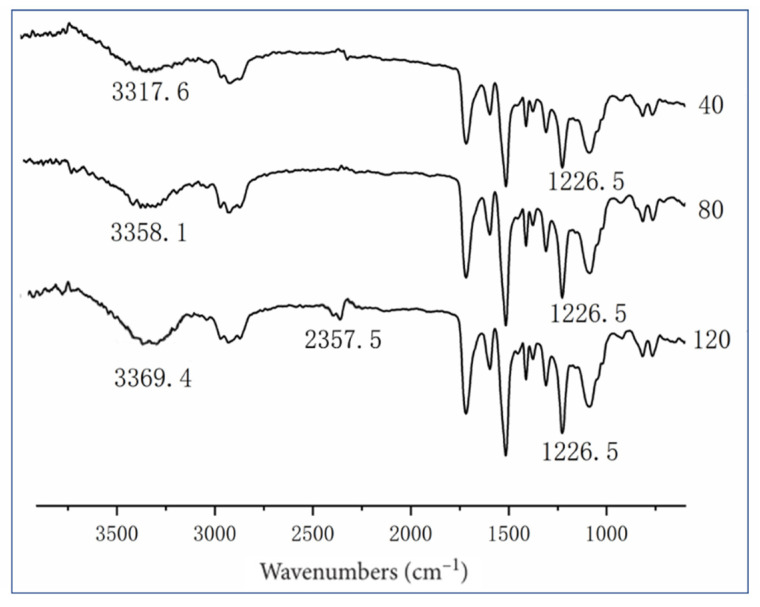
ATR-FTIR spectra of polyurethane powders with different mesh numbers.

FTIR is used to analyze the molecular structure and group changes in the degradation process, as shown in Figure 5. The characteristic peak of amino (–NH–) is 3317.6 cm^−1^ in 40 mesh. The characteristic peak of the pulverized particle size is widened in the 200 mesh, and a large amount of hydroxyl (–OH–) appeared and the characteristic peak of the large concentration of amino (–NH–) is formed by coincidence. This is the result of the C–O bond breaking to form the hydroxyl (–OH–) group. With different mesh numbers, cyanate group characteristic peaks appeared in wave numbers 3317.6–3369.4 cm^−1^, indicating the rupture of the carbamate group at the C–O bond and the emergence of a new isocyanate group.

At wavenumber 1226.5 cm^−1^, the stretching vibration peak of carbamate group C–O changed significantly, indicating that the carbamate group on the main chain of the polymerization is gradually reduced and the cross-linking structure is gradually destroyed.

#### 3.1.3. Microstructure of PUF Powder

**Figure 6:**
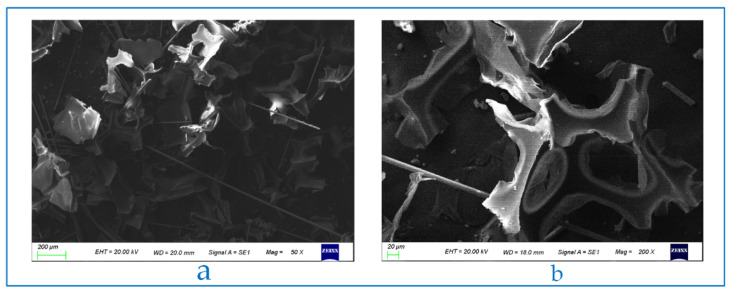
Microscopic morphology of PUF powder with different magnification. (**a**) 50 times (**b**) 200 times.

**Figure 7:**
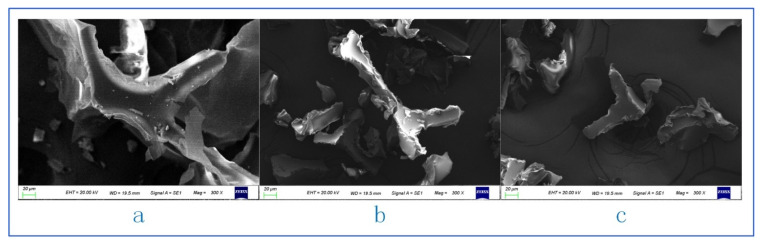
Microscopic morphology of PUF powders with different mesh numbers. (**a**) 40 mesh. (**b**) 80 mesh. (**c**) 120 mesh.

The micrograph of PUF powder is shown in Figure 6a (40 mesh, loading voltage is 20 kV, magnification is 50 times). It can be seen that there are a lot of fibers in PUF powder, which come from the fireproof layer mentioned above. In this study, choosing to retain these fibers can not only reduce the pre-treatment cost but also effectively improve the mechanical properties of plastics by adding fibers into plastics. Micrographs of 40 mesh, 80 mesh, and 120 mesh powders are shown in Figure 7 (loading voltage 20 kV, magnification is 300 times). It can be seen that the shapes of the three powders are similar, but the 40-mesh powder retains more of the pore structure of polyurethane foam, which can be seen more clearly in Figure 6b (40 mesh, loading voltage 20 kV, magnification 200 times). This explains the influence of mesh number on thermal conductivity well. The melted polypropylene covers the surface of polyurethane foam powder and generates bubbles again, which can greatly reduce the thermal conductivity. However, the existence of a large number of bubble structures also causes it to become a weak point in the tensile test.

### 3.2. Orthogonal Test Analysis

#### 3.2.1. Orthogonal Test Design

In orthogonal test design, the choice of factors and levels is very important. Based on the results of particle size analysis and infrared spectrum analysis, and the previous studies [26]. Four factors were determined, namely, mesh number (A), PUF proportion (B), temperature (C), and time (D).

(A) Mesh: After a single factor test on the mesh level, the mesh level is set to 40 mesh, 80 mesh, and 120 mesh. The larger the particle size of the powder, the more it can preserve the thermal insulation properties of the polyurethane foam itself, but too large particles will reduce the bond strength of the polyurethane foam powder and polypropylene, and the mechanical properties of the recycled board are poor. To improve the mechanical properties, a large amount of polypropylene powder is added, which makes the recycling of polyurethane foam secondary and goes against the goal. At the same time, the smaller the particle size of the powder, the better its mechanical properties. When polyurethane powder finer than 200 mesh is used and the addition amount exceeds 50%, the tensile strength of the finished product can be close to 20 Mpa. However, it has to be considered that the limited output of ultra-fine powder will greatly increase the cost of pulverization. Therefore, under the current conditions, the selection of 40 mesh, 80 mesh, and 120 mesh is more reasonable.

(B) PUF proportion: Due to the decision to recycle polyurethane foam as the main body, the proportion of polyurethane foam powder small addition was set to 50%. Considering the maximum proportion, although 100% polyurethane foam powder can be molded under high temperature and high pressure (180 °C, 35 Mpa), the molding effect is poor, and the mechanical properties are not ideal. Therefore, the maximum addition proportion of polyurethane foam is set to 80%.

(C) Temperature: The temperature is selected to make the polypropylene powder obtain fluidity. In fact, the polyurethane foam will also have a certain degree of plasticity at a certain temperature, which will help the molding of the product. Considering the melting temperature of polypropylene, a series of tests were carried out in the range of 165–215 °C. When the temperature is lower than 185 °C, the polypropylene powder has begun to flow, but the molding effect is not satisfactory. The bonding strength of the polyurethane foam powder and polypropylene is very poor, and even the powder fell off when the final product was taken. When the heating time is extended, the effect will be improved, but the mechanical properties are still not ideal and not economical enough. Therefore, it was decided to set the temperature range to 185 °C, 195 °C, and 205 °C. Under these conditions, the molding effect is the best.

(D) Time: As mentioned before, polypropylene takes time to melt and combine with the polyurethane foam powder. A series of tests were conducted in the range of 10–60 min, and it was found that 30–50 min is the most reasonable range. Too short heating time will affect the mechanical properties, and the longer time is meaningless.

Table 2 lists the details of the factors and their levels. Based on orthogonal test design table L9 (3^4^), a total of 9 groups of tests were conducted, as shown in Table 3, with each row representing one test.

#### 3.2.2. Results of Orthogonal Test

In this paper, the indexes of the orthogonal test are set as two: thermal conductivity, and tensile strength. Each set of experiments was run three times and the results were averaged. Experimental data are shown in Table 4.

### 3.3. Performance Analysis of Board

#### 3.3.1. Thermal Conductivity Analysis

**Figure 8:**
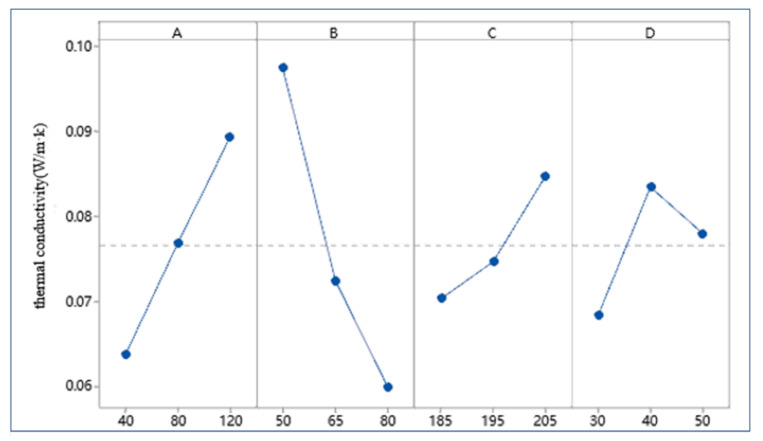
Effect of factors at different levels on thermal conductivity: (**A**) Mesh. (**B**) Proportion. (**C**) Temperature. (**D**) Time.

Table 5 shows the results of the range analysis of thermal conductivity. K_i_ represents the average value of thermal conductivity under a certain factor. The mesh size (A) is positively correlated with the thermal conductivity. The larger the mesh size of the powder is, the larger the thermal conductivity is, that is to say, the worse the thermal insulation performance is (Figure 8A). Temperature (C) showed similar results to mesh (A) (Figure 8C). On the contrary, the higher the proportion of polyurethane powder (B), the lower the thermal conductivity (Figure 8B). When the value of factor time (D) increases, the corresponding value of Ki increases first and then decreases (Figure 8D). According to R-value, the factors can be arranged as B > A > D > C, indicating that the proportion of polyurethane has the greatest influence on thermal conductivity, followed by the particle size of polyurethane powder, temperature and time have less influence.

**Figure 9:**
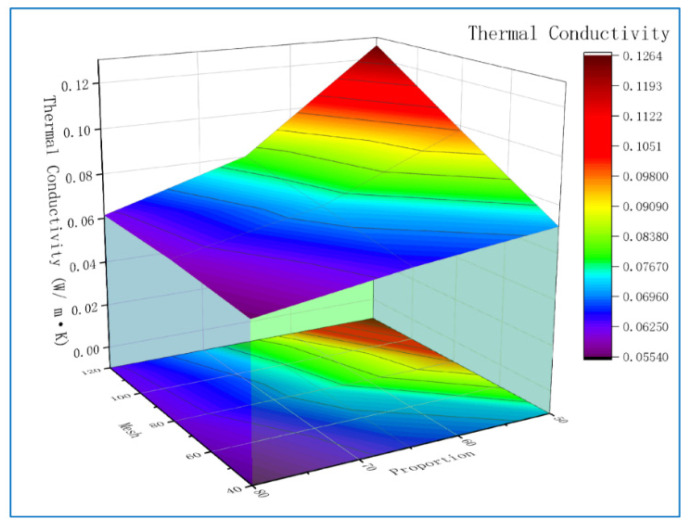
The surface projection of Mesh and Proportion affects thermal conductivity.

According to the results in Table 4, ANOVA is conducted for thermal conductivity, and statistical results were listed in Table 6. SS is the sum of squares of variables; DOF represents degrees of freedom; MS is mean square, that is, the ratio of SS to DOF; F and P are the values that determine whether the variable is significant. The high value of F and the low value of P indicate that the variable is more significant [27]. As can be seen from Table 6, the results of ANOVA are consistent with the previous range analysis, and the proportion of polyurethane powder has an important influence on the thermal conductivity. Because polyurethane foam powders do not melt when heated, there are many gaps between the powders, and the melted polypropylene seeps into these gaps and joins the powders. As the proportion of polyurethane foam powder increases, the proportion of polypropylene decreases, and polypropylene cannot be filled into the gap, resulting in a large number of bubbles. This is also the reason why the mesh size will affect the thermal conductivity. The smaller the mesh of the powder, the larger the particle size, and the gap is also larger. This can also be seen in the micrograph. To more intuitively express the influence of factors on thermal conductivity, the proportion and mesh number of polyurethane foams are selected as conditions to draw the Surface projection, as shown in Figure 9. Minitab software is used to analyze the linear regression equation of thermal conductivity coefficient *Υ*_1_:

*Υ*_1_ = −0.0233 + 0.000320A − 0.001323B + 0.0023C + 0.000482D (2)

#### 3.3.2. Tensile Strength Analysis of Board

**Figure 10:**
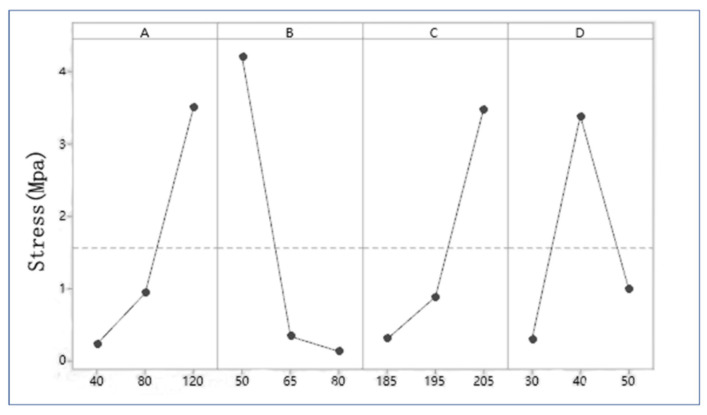
Effect of factors at different levels on stress: (**A**) Mesh. (**B**) Proportion. (**C**) Temperature. (**D**) Time.

Table 7 shows the results of the range analysis of tensile strength. According to R-value, the order of factor influence is: B > A > C > D. It can be seen that the influence trend of different factors on tensile properties is consistent with the thermal conductivity, as shown in Figure 10.

**Figure 11:**
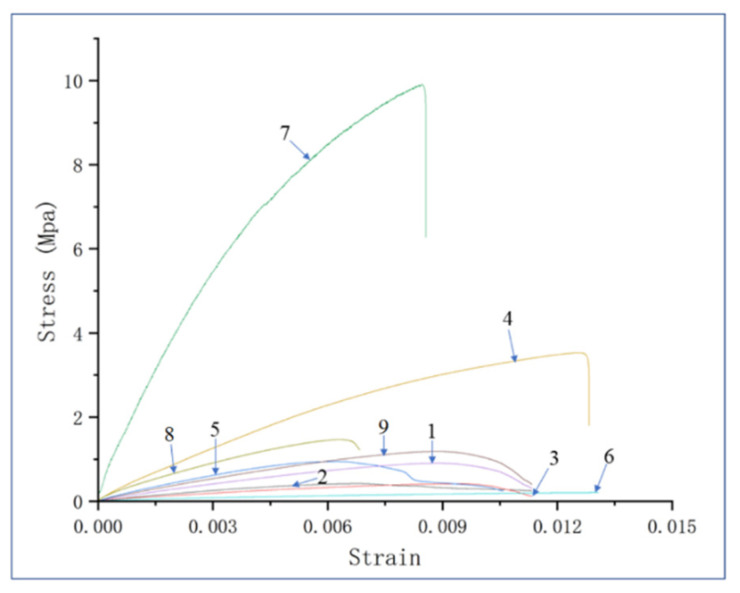
Stress versus strain curve (The numbers in the figure represent different experiment numbers).

ANOVA is conducted for tensile strength, and the results were shown in Table 8. Mesh, proportion, temperature, and time have significant effects on the tensile strength, and the ranking of their contribution degree is consistent with the range analysis results. To observe the tensile properties more intuitively, the stress-strain data are drawn, as shown in Figure 11. It can be seen that the stress of the No.7 broad is much higher than that of other broads. From its processing parameters (120 mesh, 50%, 205 °C, 40 min), this result is inevitable. 120 mesh PUF powder has stronger surface activity and can be better combined with PP powder. The high proportion of PP powder provides a higher tensile strength for the No.7 broad. The temperature of 205 °C, and the time of 40 min, provide the possibility of a good combination of PUF and PP. The second highest stress is no.4 broad, whose machining parameters are (80 mesh, 50%, 195 °C, 50 min). Comparing the thermal conductivity and tensile strength of these two boards, it can be seen that they have the highest values of both. High tensile strength means better powder bonding and lower porosity. This also makes the thermal conductivity higher.

**Figure 12:**
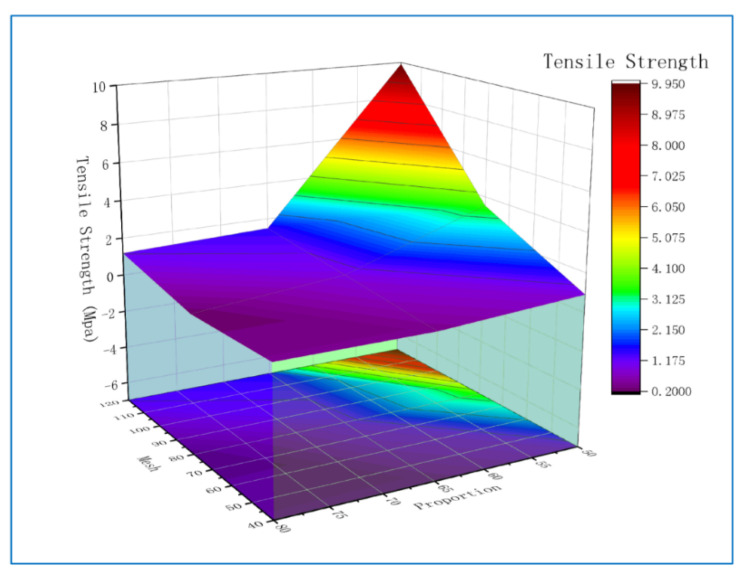
The surface projection that Mesh and Proportion affect tensile strength.

Similarly, the proportion and mesh number of polyurethane foams are selected as conditions to draw the surface projection of its influence on tensile strength, as shown in Figure 12. The linear regression equation of tensile strength *Υ*_2_ is:

(3) $$\Upsilon_{2}=\;-22.37\;+\;0.04524A-0.1392B\;+\;0.1392C\;+\;0.0390D$$

#### 3.3.3. Microstructure of Recycled Board

**Figure 13:**
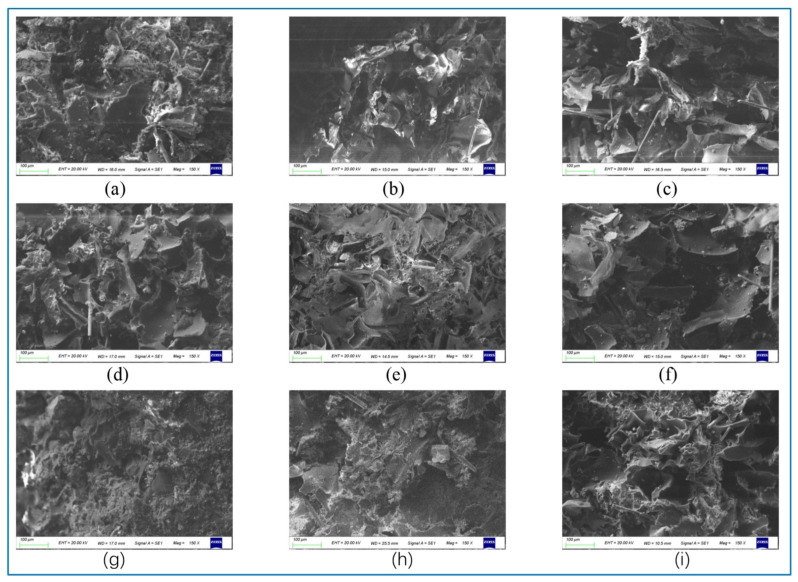
Photomicrographs for samples 1–9 (×150 magnification; (**a**–**i**) corresponds to experiments no. 1–9).

Micrographs of nine boards are shown in Figure 13. The fireproof layer fibers are evident in Figure 13c,f. Note that each row has the same number of mesh and each column has the same proportion. Therefore, it is also easy to compare the effects of mesh and proportion on the board. The lowest thermal conductivity is board No. 3, which can also be seen by comparing 13c with other horizontal and longitudinal pictures. A large number of polyurethane foam powders provides the possibility of low thermal conductivity. A small amount of polypropylene cannot completely wrap the polyurethane foam powder and only plays a role of connection, which is also the reason why the tensile strength of No. 3 board is only 0.4143 Mpa. The opposite is board No. 7, whose micrograph is shown in 13 g. Board No. 7 contains 50%, 120 mesh polyurethane foam powder, which is completely coated with equal weight polypropylene. It is not available on other boards. It also brings excellent tensile strength to the No. 7 plate. It is worth mentioning that the thermal conductivity of No. 7 board is 0.1263 W/m·K, which is 54% of the thermal conductivity of pure polypropylene board (about 0.23 W/m·K). It is also proved that 120 mesh polyurethane powder has a great influence on reducing thermal conductivity.

#### 3.3.4. Parameter Selection of Recycled Board

Based on the above analysis, we can see that the thermal conductivity is positively correlated with the tensile strength, which makes it difficult to obtain an optimal solution and needs to be adjusted according to actual requirements. This study provides three parameters for reference: the lowest thermal conductivity, the maximum tensile strength, and the equilibrium selection.

The lowest thermal conductivity is selected as A_1_B_3_C_1_D_1_. Under this condition, the thermal conductivity is 0.037 W/m·K, the tensile strength is 0.133 Mpa. The low strength makes it difficult to use as a stand-alone material. However, it can be used as the building insulation boards, surrounded by brick, concrete, reinforced concrete, and other heavy materials. Or as the sandwich of steel board, to provide better insulation ability.

The maximum tensile strength was selected as A_3_B_1_C_3_D_2_. Under this condition, the thermal conductivity was 0.1253 W/m·K, the tensile strength was 9.913 Mpa. Its thermal insulation performance is general, but good mechanical properties can be used for room decoration panels, pipes, bumpers, gaskets.

In addition, according to range analysis, the influence of proportion (B) on the thermal conductivity is much higher than the other three factors. Although the proportion (B) has the highest influence on tensile strength, the difference between it and the other three factors is small. Therefore, B_3_ is selected to obtain better thermal insulation performance, and A_3_C_3_D_2_ is selected to obtain better tensile strength. Under the condition of A_3_B_3_C_3_D_2_, the thermal conductivity is 0.086 W/m·K, the tensile strength is 5.737 Mpa. Balanced performance can be applied to a wide range of uses, such as replacing lightweight aggregate concrete for the interior and exterior walls of buildings, roofs, and floors.

For comparison, the above results and the properties of the original material are listed in Table 9. It can be seen that the thermal conductivity is as low as 0.037 W/m·K, which is very close to the thermal conductivity of polyurethane foam. In the past research on thermosetting plastics, researchers focused on the mechanical properties of recycled plates. For example, Prestes et al. [14] Added 40% high-pressure Laminate powders into polypropylene, extruded by the extruder model and the tensile strength was 11.58 Mpa. Quadrini et al. [28] formed pure polyurethane foam powders by hot pressing. The tensile strength and compressive strength were 2.4 Mpa and 22 Mpa respectively. Considering only the mechanical properties, it is undoubtedly a waste of polyurethane foam with high insulation capacity. In terms of the highest thermal conductivity, the value of 0.1253 W/m·K is 54% of the thermal conductivity of pure polypropylene board, which has also met the requirements of China for thermal insulation materials. Its 9.913 Mpa tensile strength far exceeds that of polyurethane foam. Taking into account that under A_3_B_1_C_3_D_2_ condition, the proportion of polyurethane foam powder is 50%, the mechanical properties of this recycled board are not weaker than the study by Prestes et al.

## 4. Conclusions

Following are the conclusions from this study:

The effect of mechanochemical pulverization of waste polyurethane foam on the appearance and molecular structure of PUF is studied. As a result, the mechanical and thermal energy is accumulated during a long period of crushing. Under the combined action, the C–O bond of PUF is broken, the network crosslinking structure is destroyed, and the activity of PUF powder is significantly improved.

Polyurethane foam powder and PP can be remolded into composite materials by heat pressing. Taking mesh, proportion, temperature, and time as factors and thermal conductivity, tensile strength, and density as indexes, the orthogonal test design method is established. Based on range analysis and variance analysis, the influence of each factor on the index is studied.

The results show that the proportion of polyurethane foam powder has the greatest influence on thermal conductivity and tensile strength, the second is mesh size, and the temperature and time have less influence. When the mesh number is 40 and the proportion is 80%, the lowest thermal conductivity 0.037 W/m·K and the tensile strength is 0.133 Mpa are obtained. The polyurethane foam powder at the age of 40 mesh retains a relatively complete bubble structure, but the melting of 20% polypropylene is not enough to fill it but will form new and smaller bubbles. When the mesh number is 120 and the proportion is 50%, the maximum tensile strength is 9.913 Mpa and the thermal conductivity is 0.1253 W/m·K. However, the value of 0.1253 W/m·K, which is 54% of the thermal conductivity of pure polypropylene board (about 0.23 W/m·K), has also reached the requirements of China for thermal insulation materials.

This study provides an effective method for the recovery of polyurethane foam, and as an example of applications can be expected: Insulation, roofs, bumpers, gaskets, etc. More research is needed to improve the properties of recycled polyurethane foam. The performance of the recycled boards was slightly worse than that of the original material, which is to be expected considering that no additives were added in this test. In the next test, additives will be selected and different thermoplastics will be tried.

## Figures and Tables

**Table 1:** Categories of PU applications.

Categories	Applications	Production
Flexible foams	Furniture, carpet, bedding, matrasses	36%
Rigid foams	Commercial refrigerators, insulation board, packaging	32%
Elastomers	Implants, medical devices, shoe soles	8%
Adhesives and sealant	Casting, sealants	6%
Coatings	Aircraft, vehicles (bumpers, side panels)	14%
Binders	Assembling of wood boards, rubber, elastomeric flooring surfaces	4%

**Table 2:** The parameters and levels for processing.

Levels	A (Mesh)	B (Proportion)	C (Temperature)	D (Time)
1	40	50%	185	30
2	80	65%	195	40
3	120	80%	205	50

**Table 3:** DOE for final experimentation.

Exp No.	Mesh	Proportion	Temperature (°C)	Time (min)
1	40	50%	185	30
2	40	65%	195	40
3	40	80%	205	50
4	80	50%	195	50
5	80	65%	205	30
6	80	80%	185	40
7	120	50%	205	40
8	120	65%	185	50
9	120	80%	195	30

**Table 4:** Orthogonal scheme and its results.

Test	A	B	C	D	Thermal Conductivity (W/m·K)	Tensile Strength (Mpa)
1	1	1	1	1	0.0711	0.9031
2	1	2	2	2	0.0645	0.4182
3	1	3	3	3	0.0555	0.4143
4	2	1	2	3	0.0982	3.5275
5	2	2	3	1	0.0726	0.9451
6	2	3	1	2	0.0596	0.2177
7	3	1	3	2	0.1263	9.9129
8	3	2	1	3	0.0803	1.4642
9	3	3	2	1	0.0614	1.1847

**Table 5:** Range analysis of thermal conductivity.

Elements	A	B	C	D
K_1_	0.0637	0.0985	0.0703	0.0684
K_2_	0.0768	0.0725	0.0747	0.0835
K_3_	0.0893	0.0588	0.0848	0.078
R	0.0256	0.0397	0.0145	0.0151

**Table 6:** ANOVA result for the thermal conductivity.

Variable	SS	DOF	MS	F	*p*	Contribution
A	0.002957	2	0.001479	25.64	<0.001	23.99%
B	0.007324	2	0.003662	63.51	<0.001	59.42%
C	0.000991	2	0.000496	8.59	0.002	8.04%
D	0.001052	2	0.000526	9.12	0.002	8.54%
Error	0.001038	18	0.000058			
Total	0.013363	26				100%

**Table 7:** Range analysis of tensile strength.

Elements	A	B	C	D
K_1_	0.5544	4.7811	0.8616	1.0109
K_2_	1.5634	0.9183	1.6860	3.4921
K_3_	4.1872	0.6055	3.7574	1.8020
R	3.6328	4.1755	2.8958	2.4811

**Table 8:** ANOVA result for the tensile strength.

Variable	SS	DOF	MS	F	*p*	Contribution
A	63.148	2	31.5739	66.68	<0.001	27.43%
B	96.860	2	48.4300	102.28	<0.001	42.08%
C	40.309	2	20.1545	42.56	<0.001	17.51%
D	29.870	2	14.9349	31.54	<0.001	12.98%
Error	8.523	18	0.4735			
Total	238.710	26				100%

**Table 9:** Performance and application of the board.

Parameter	Thermal Conductivity (W/m·k)	Tensile Strength (Mpa)	Applications
A_1_B_3_C_1_D_1_	0.037	0.133	Insulation board, the sandwich of steel broad
A_3_B_1_C_3_D_2_	0.1253	9.913	Room trim panels, pipes, bumpers, gaskets,
A_3_B_3_C_3_D_2_	0.086	5.737	Walls, roofs, floors
Polyurethane foam	0.022~0.030	0.3	
Polypropylene	0.23	29	

## Data Availability

Data generated or analyzed during this study are included in this published article.

## References

[1] Wang M., Zhang X., Zhang W., Lu C., Yuan G. (2014). From Thermosetting to Thermoplastic: A Novel One-Pot Approach to Recycle Polyurethane Wastes via Reactive Compounding with Diethanolamine. Prog. Rubber Plast. Recycl. Technol..

[2] Plastics Europe Association of Plastics Manufacturers Plastics—The Facts 2019 an Analysis of European Plastics Production, Demand and Waste Data. https://www.plasticseurope.org/en/resources/market-data.

[3] Deng Y., Dewil R., Appels L., Ansart R., Baeyens J., Kang Q. (2021). Reviewing the thermo-chemical recycling of waste polyurethane foam. J. Environ. Manag..

[4] Stachak P., Lukaszewska I., Hebda E., Pielichowski K. (2021). Recent Developments in Polyurethane-Based Materials for Bone Tissue Engineering. Polymers.

[5] Magnin A., Pollet E., Phalip V., Averous L. (2020). Evaluation of biological degradation of polyurethanes. Biotechnol. Adv..

[6] Yang H., Yu B., Song P., Maluk C., Wang H. (2019). Surface-coating engineering for flame retardant flexible polyurethane foams: A critical review. Compos. Part B Eng..

[7] Yang W., Dong Q., Liu S., Xie H., Liu L., Li J. (2012). Recycling and Disposal Methods for Polyurethane Foam Wastes. Procedia Environ. Sci..

[8] Wang B., Ma S., Li Q., Zhang H., Liu J., Wang R., Chen Z., Xu X., Wang S., Lu N. (2020). Facile synthesis of “digestible”, rigid-and-flexible, bio-based building block for high-performance degradable thermosetting plastics. Green Chem..

[9] Yuan Y., Sun Y., Yan S., Zhao J., Liu S., Zhang M., Zheng X., Jia L. (2017). Multiply fully recyclable carbon fibre reinforced heat-resistant covalent thermosetting advanced composites. Nat. Commun..

[10] Tantisattayakul T., Kanchanapiya P., Methacanon P. (2018). Comparative waste management options for rigid polyurethane foam waste in Thailand. J. Clean. Prod..

[11] Kemona A., Piotrowska M. (2020). Polyurethane Recycling and Disposal: Methods and Prospects. Polymers.

[12] Gharde S., Kandasubramanian B. (2019). Mechanothermal and chemical recycling methodologies for the Fibre Reinforced Plastic (FRP). Environ. Technol. Innov..

[13] Singh R., Singh I., Kumar R., Brar G. (2019). Waste thermosetting polymer and ceramic as reinforcement in thermoplastic matrix for sustainability: Thermomechanical investigations. J. Thermoplast. Compos. Mater..

[14] Prestes P., Domingos M., Faulstich D. (2019). Effect of high pressure laminate residue on the mechanical properties of recycled polypropylene blends. Polym. Test..

[15] Yang C., Zhuang Z., Yang Z. (2014). Pulverized polyurethane foam particles reinforced rigid polyurethane foam and phenolic foam. J. Appl. Polym. Sci..

[16] Gama N.V., Ferreira A., Barros-Timmons A. (2018). Polyurethane Foams: Past, Present, and Future. Materials.

[17] Moon J., Kwak S., Lee J., Kim D., Ha J., Oh J. (2019). Synthesis of polyurethane foam from ultrasonically decrosslinked automotive seat cushions. Waste Manag..

[18] Gama N., Godinho B., Marques G., Silva R., Barros-Timmons A., Ferreira A. (2021). Recycling of polyurethane by acidolysis: The effect of reaction conditions on the properties of the recovered polyol. Polymers.

[19] Godinho B., Gama N., Barros-Timmons A., Ferreira A. (2021). Recycling of different types of polyurethane foam wastes via acidolysis to produce polyurethane coatings. Sustain. Mater. Technol..

[20] Valle V., Aguirre C., Aldás M., Pazmiño M., Almeida-Naranjo C. (2020). Recycled-based thermosetting material obtained from the decomposition of polyurethane foam wastes with castor oil. J. Mater. Cycles Waste Manag..

[21] Heiran R., Ghaderian A., Reghunadhan A., Sedaghati F., Thomas S., Haghighi A. (2021). Glycolysis: An efficient route for recycling of end of life polyurethane foams. J. Polym. Res..

[22] Gama N., Godinho B., Marques G., Silva R., Barros-Timmons A., Ferreira A. (2020). Recycling of polyurethane scraps via acidolysis. Chem. Eng. J..

[23] James S., Adams C., Bolm C., Braga D., Collier P., Friscic T., Grepioni F., Harris K., Hyett G., Jones W. (2012). Mechanochemistry: Opportunities for new and cleaner synthesis. Chem. Soc. Rev..

[24] Zhang C., Zhuang L., Yuan W., Wang J., Bai J. (2016). Extraction of lead from spent leaded glass in alkaline solution by mechanochemical reduction. Hydrometallurgy.

[25] Hu J., Dong H., Song S. (2020). Research on Recovery Mechanism and Process of Waste Thermosetting Phenolic Resins Based on Mechanochemical Method. Adv. Mater. Sci. Eng..

[26] Wu W., Liu G., Cheng H. (2016). Preparation and Performance Analysis of Regenerated Materials for Thermosetting Polyurethane Based on Coupled Thermo-mechanical Model. Chin. J. Mech. Eng.-Engl. Ed..

[27] Li H., Xu B., Lu G., Du C., Huang N. (2021). Multi-objective optimization of PEM fuel cell by coupled significant variables recognition, surrogate models and a multi-objective genetic algorithm. Energy Convers. Manag..

[28] Quadrini F., Bellisario D., Santo L. (2013). Recycling of thermoset polyurethane foams. Polym. Eng. Sci..

